# Exploring the feasibility and acceptability of the contents, design, and functionalities of an online intervention promoting mental health, wellbeing, and study skills in Higher Education students

**DOI:** 10.1186/s13033-019-0308-5

**Published:** 2019-07-23

**Authors:** Marietta Papadatou-Pastou, Lauren Campbell-Thompson, Elizabeth Barley, Mark Haddad, Caroline Lafarge, Eamonn McKeown, Louise Simeonov, Patapia Tzotzoli

**Affiliations:** 1 School of Education, Faculty of Primary Education, National and Kapodistrian University of Athens, 13A Navarinou Str, 106 80 Athens, Greece; 2 iConcipio Ltd, South London and Maudsley, Camberwell, London, SE 5 8 AZ UK; 3 University of West London, Paragon House, PR405, Boston Manor Road, Brentford, Middlesex TW8 9GA UK; 4 City, University of London, London, UK; 5 School of Human and Social Sciences, University of West London, Paragon, Boston Manor Road, Brentford, TW8 9GA UK; 6 University College London, 1-19 Torrington place, London, UK; 7 My Psychology Clinic and iConcipio Ltd, 13 Orchard Rise, Richmond, Greater London, TW10 5BX UK

**Keywords:** Students, Mental health, Higher education, Online intervention, MePlusMe, Wellbeing

## Abstract

**Background:**

Substantial numbers of students in Higher Education (HE) are reporting mental health difficulties, such as mild to moderate symptoms of depression and anxiety. Coupled with academic skills challenges, these difficulties can lead to decreased academic performance, low levels of study satisfaction, and eventually drop out. Student support services are facing budget cuts and can only attend to limited numbers of students, usually the ones who present with more severe mental health problems. Moreover, face-to-face contact may not appeal to those students who feel embarrassed by their problems or are afraid of being stigmatised. To address this important problem, an online psychological wellbeing and study skills support system called MePlusMe, has been developed to provide personalised support to its users. In the present study we investigated the feasibility and acceptability of the contents, design, and functionalities of the system.

**Methods:**

An offline version of the system was introduced to 13 postgraduate and undergraduate students (mean age = 31.3 years, SD = 10.25 years; 4 males) in a UK HE Institution, who presented with mild or moderate mental health difficulties. The participants evaluated the design of the system, its functionalities, and contents at Baseline and at Weeks 2, 4, and 8.

**Results:**

Participants found the system easy to use, professional, and efficient and its contents non-judgemental and informative. Participants stated that engaging with and practicing the techniques targeted at mental health difficulties led to improvements in positive thinking and self-confidence, while the study skills techniques were practical. Suggestions for further improvement included the development of an app and an option for direct engagement with professionals.

**Conclusions:**

The findings confirmed the acceptability of the contents, design and functionalities of the system, while providing useful information to inform its further development. Next steps include a feasibility study, which will test and quantify the effects on everyday functioning, mood, mental wellbeing, and academic self-efficacy after using the system, and subsequently a randomized controlled trial, which will evaluate its effectiveness.

The life of a Higher Education (HE) student can come with a wealth of exciting experiences, invaluable memories, and new challenges. However, during such a critical period of personal, social, and academic development, some of these new challenges may result in initiating or exacerbating existing mental health issues or in making study skills challenges more prominent [1]. A worrying number of students have indeed been found to experience mild to moderate symptoms of depression or anxiety, with the number of students who experience mental health difficulties whilst at university increasing [2]. An online system for psychological, as well as academic, support has been designed to specifically address the growing needs of HE students, MePlusMe. Here we present evidence for the feasibility and acceptability of the system’s contents, design, and functionalities.

## Background

An increasing number of students in Higher Education Institutions (HEIs) are experiencing mental distress and mental health conditions in the UK. In 2015 the Higher Education Statistics Agency (HESA) reported that a total of over eighty thousand students requested counselling from their HEI’S, compared to sixty thousand in 2013, a rise of over 27% [3]. YouGov’s 2016 national survey found that one in four students suffer with mental health issues, with 77% experiencing depression-related symptoms and 74% experiencing anxiety-related symptoms [2]. More recently, the World Health Organisation (WHO) World Mental Health International College Student Initiative reported that one in three first year university students experience symptoms of a mental health condition [4]. Furthermore, the WHO contributed to a study at Ulster University in Northern Ireland to monitor student wellbeing using the WHO World Mental Health Composite International Diagnostic Interview (WMH-CIDI). Results showed high baseline prevalence rates of both short-term and long-term mental health and substance disorders, Attention-Deficit/Hyperactivity Disorder (ADHD) and suicidality, with more than 50% of new undergraduate students reporting a lifetime disorder. Alarmingly, co-morbidity was common with 19.1% of students experiencing three or more disorders at a given time [5].

In 2014 HESA surveyed 2843 students and found that the prevalence of depression and anxiety was 15.6% among undergraduate students (13% for graduate students) [6]; whilst internationally a meta-analysis involving 24 studies conducted in the USA, UK, EU, and other nations identified a weighted mean depression prevalence of 30.6% [7], although these studies all used validated self-report measures rather than diagnostic interview methods.

Anxiety and depression symptoms are the most commonly reported by HE students [8]; with over 77% of students reporting depression-related symptoms, 74% reporting anxiety-related symptoms, and a 74% co-incidence rate [2]. The Royal College of Psychiatrists (RCP) found that students are more likely to experience mental health difficulties or symptoms compared to age-matched peers outside of HE [9]. Worryingly, these findings may underestimate the true scope of the issue due to the social stigma surrounding mental health difficulties or due to such difficulties being undetected or unreported [10]. Indeed, studies have shown that although student support services are frequently advertised by HEI’s, many students are reluctant to seek support [9, 11] or avoid doing so [12]. A national survey conducted in 2013 on behalf of the National Union of Students (NUS) reported that 8% of students identified themselves as “having a mental health problem but not seeking diagnosis” [13] with 10% reporting having been diagnosed but not actively seeking treatment. Within the United States, a recent study found using the 12-item general health questionnaire [14] that 32% of doctoral students are at risk of having or developing a common psychiatric disorder, with the most common being depression [15].

The effects of mental distress and untreated mental health conditions can be debilitating, and has been highlighted in HE students in the form of decreased levels of academic performance [16]. Study skill problems and poor psychological wellbeing, independently and in conjunction have been found to negatively impact academic potential, decrease levels of engagement, lower graduation rates, and increase academic dropout rates [2, 17]. HESA has reported substantial numbers of students experiencing study skill difficulties i.e. over 90% of students reported issues with exam stress and deadline stress [3]. Another recent study found that 92% of students attending university counselling sessions were having problems completing their academic work [18]. For international students, other study skill difficulties may present themselves. For example, a big challenge for international students concerns studying in a non-native tongue. Although there is a minimum language requirement for HE course entry, even when students do meet the entry criteria they may not be familiar with technical terminology for a specialist subject area. This has been found to be problematic for some international students at the initial stages of a course and during an already pressured time [19].

### Student support services

HEIs often provide on-site student support services which may include academic services, such as essay writing courses, employment services, such as Curriculum Vitae (CV) workshops, and on site counselling. Data requested under the Freedom of Information Act shows that between 2011 and 2016 there was an 84% increase in the number of students contacting the counselling support service at their HEI [20]. A 94% increase was found by the Institute for Public Policy Research (IPRR) [21]. Moreover, 67% of HEIs were found not to be able to provide students access to NHS mental health specialists who can deliver interventions onsite and 23% not to work closely with NHS secondary mental health services [21]. Financial pressures placed on HE students due to government funding decreases in particular courses, such as nursing, as well as high student to lecturer ratios have led to an increased demand for study support needs. For example, the National Student Loans Company provisional data for the academic year of 2016/17 showed a decrease of almost £0.6 billion or 36% was awarded based on the previous year [22]. Early data shows that for 2017/18 a further fall of £0.55 billion is to be expected [23]. Other possible explanations for the increased numbers in HE students experiencing mental health difficulties are the removal of protective factors, for example, larger class sizes compared to those in high school can make it more difficult to socialise for some, and increased demands on academic staff can result in less individual support for students. Another explanation for increased demand could be the 2016 British government Widening Access Scheme, which aimed to encourage students from wider backgrounds to apply for HE and indeed resulted in a steady 2% increase in university applications between 2015/16 and 2016/17 [24].

In 2011 the RCP reported that access to mental health services on the NHS has progressively narrowed down to focus on high-intensity treatments and severe illnesses, resulting in the clear majority of students presenting with mild to moderate symptoms not fitting the criteria for NHS Primary Mental Health Care [9]. This narrowing of focus is the result of an influx of self-referrals for primary mental health care services from the general population to the NHS [25] due to increased accessibility and awareness. The effect of this increasing demand on HEIs’ student counselling services has been substantial. Students are often left without adequate support as student to counsellor ratios are typically less than favourable [26]. Students frequently report response times to their initial enquiries at up to 2-weeks [9] and up to 9 weeks from referral to assessment [27]. Research suggests that long waiting times can result in poorer mental health outcomes, such as more days in inpatient care and longer recovery times [28].

Another limitation of student support services is the lack of continuity of care that students may face when using them [29]. For example, many students now study away from home and internationally, leaving them without support outside of term time. It is therefore imperative that a flexible solution is found.

### Online support

The RCP [9] suggests self-help programmes and guides such as web-based interactive cognitive behavioural therapy (CBT) for non-emergency situations, leaving face-to-face counselling prioritized for those with a severity of distress, disabilities and academic difficulties. The use of these tools will likely increase the number of those seeking diagnosis and treatment [30] as well as improve standards of treatment [31, 32] and continuity of care and reduce dropout rates in HEI’s and possibly improve academic grades [33].

There is substantial evidence that supports the effectiveness of computer-based programmes when compared to face-to-face CBT [31, 34, 35]. In addition to this, groups that may be hard to contact on a face-to-face basis, such as those with anxiety disorders and depression, may particularly benefit from online CBT [36]. Computerised programmes and application-based CBT also enable the user to maintain anonymity and privacy, avoid being subjected to long waiting lists and removing the stigma that surrounds appointments with a counsellor [37]. Recent studies have shown great progress with the use of online support systems as interactive interventions, demonstrating their positive impact on accessibility and their flexibility [38, 39]. Furthermore, effects have been shown to be longitudinal, with self-reported symptoms significantly reduced 12 months post participation [40, 41].

In recent years, several web-based systems have started to offer psychological support, advice, and information to the public, for example NHS Silvercloud and PLUS [42]. However, most of these systems focus on the general population and only a few address the study skill issues that HE students face. The few systems that target HE students, for example, “CALM” (Computer Aided Lifestyle Management) and “Students Against Depression”, have yet to be tested for their feasibility or effectiveness and unfortunately do not address study skills [42]. Moreover, these systems offer pre-made, non-tailored packages for specific conditions.

The present paper will investigate the feasibility and acceptability of the contents, design, and functionalities of MePlusMe, an online support system designed specifically for HE students who are facing mild to moderate psychological and/or study skill difficulties, or for students who just simply want to take care of their psychological wellbeing and improve their academic competence. MePlusMe is the only system that currently offers personalised interventions in video format for HE students by addressing depression and anxiety symptoms and study skill difficulties. Unlike these, the packages offered by MePlusMe can vary each time depending on the user’s present difficulties, thus facilitating multiple uses from the same user, resulting in an increased likelihood of recurrent and long-term engagement.

### MePlusMe

iConcipio has designed a web-based solution under the name MePlusMe [43]. It is designed to help students with mild to moderate mental health and/or study skills difficulties, as well as students who do not present with any specific difficulties but who desire to learn how to take care of their psychological wellbeing and improve their study skills. MePlusMe can be used as a stand-alone tool or alongside traditional face-to-face services.

Several filters throughout the system, including a “panic button”, refer students with severe difficulties to other services for more intensive support. The rest of the students can easily use the system by following one of the two available routes. The first is a symptoms-based assessment (Questionnaire route) that invites users to identify the symptoms they experience, and the second is a technique-driven approach (Library route) whereby users select directly their preferred techniques. Both routes lead to a customised package of psychological wellbeing and/or study skill techniques presented in 2D animated video format.

The screening questionnaire has been adapted from the following established tools and clinical questionnaires: the Hospital Anxiety and Depression Scale [HADS] [44]; the Generalized Anxiety Disorder Scale [GAD-7] [45]; the Patient Health Questionnaire [PHQ-9] [46]; and the Mini International Neuropsychiatric Interview [M.I.N.I.] [47]. The HADS, GAD-7, and M.I.N.I. formed the choice of anxiety symptom-based questions. The HADS, PHQ-9, and M.I.N.I. formed the choice of depression symptom-based questions.

The design of the questionnaire addresses symptoms, instead of conditions/diagnoses and the system automatically links clusters of symptoms with specific video techniques. As a result, each package represents the best-fit solution for students tailored to address the specific difficulties they face each time. The library route leads to the package of techniques that students themselves see as best-fit to address their challenges. This route allows students the flexibility to edit their packages at any point by adding the techniques that they prefer or by deleting unsuitable techniques.

All the techniques that are provided are evidence-based. The psychological techniques derive from current treatment models such as Cognitive Behavioural Therapy (CBT) and Mindfulness [48–50] and the study skills techniques include strategies such as how to stay motivated and manage time effectively. The use of multimedia has been suggested to facilitate the active process of learning [51], which is why the techniques are presented in a relatable animated audio-visual format accompanied by downloadable printouts. The package of techniques that are to be practiced by the user is stored on the user’s “MyPlace”. Students can login and watch the videos anytime and from anywhere they wish. A reminder option that users are encouraged to make use of and which sends emails prompting them to return and practise their techniques within a period of 8 weeks, is also available.

Users are also asked to report how well they are doing over time, starting on the day they undertake the Questionnaire or Library route and then after 2, 4, and 8 weeks. This self-monitoring progress is shown in the form of a motivational graph. When a package is not relevant anymore, students can archive it for later use. Moreover, they can quickly access and restore past packages any time they wish in order to use them again. MePlusMe further offers an integrated, monitored online peer support network. Student engagement is encouraged in this social section of the site, called “Thoughtwall”, a space where students can post their thoughts under their chosen nickname, “like” the shared thoughts of other users, and share their progress graph after completion of a package. They can also share their thoughts and graphs to other sites outside MePlusMe. Finally, students can personalise their profile by uploading pictures of their preference on their “Wall”.

### System development

Preliminary market research conducted via the use of semi-structured interviews with counsellors and psychologists working in student support services within four UK HEIs (London School of Economics, King’s College London, University College London, and Kingston University) revealed current challenges and positive responses to the enquiry about an online solution (Tzotzoli, personal communication, 2011). This step enabled researchers to gather an understanding of current support services, the challenges they are facing, and whether an online system could fit into the market. An online survey was subsequently conducted which helped to identify difficulties faced by students at university, opinions on online support systems and what features students may want the system to include, or what may look appealing on the site [52]. Findings demonstrated a demand and space in the market for an online system, and they further highlighted student needs and system requirements. iConcipio was awarded a Proof of Concept Grant (Smart Award) which allowed a beta version of MePlusMe to be constructed to demonstrate the system during a proof-of-concept study [53]. With the help of a cohort of 873 students from five UK HEI (King’s College London, University of Warwick, University of Edinburgh, Bournemouth University, and University of Roehampton) the proof-of-concept study confirmed the conceptual and practical value (suitability) of MePlusMe. Feedback was collected from students about the main aspects of the proposed design, system contents, aesthetics, and the process of delivery. The results from this study were then used for system refinement. Members of an Academic Advisory Board and a Research Advisory Board, consisting of clinical psychologists and academics have further ensured that MePlusMe’s design and contents adhere to best psychological practice and supervised this work.

### Scope of the present study

To date, iConcipio has developed the contents of MePlusMe, namely the design and all the initial video techniques and certain functionalities of the platform, with the exception of some automatized ones. The present study aims to collect qualitative data on the feasibility and acceptability of the MePlusMe’s contents in order to further develop the system. Furthermore, data gained from participants will contain user feedback on how engaging they found the media elements of MePlusMe (the video techniques). It will introduce this material offline to UK HEI students who will be administered one of the routes (Questionnaire or Library) and who will then receive their own customised package of techniques. Students will answer questions regarding the system’s design, functionalities, and video contents. They will then be advised to use their suggested techniques on an as and when needed basis. The students will be approached again to answer questions regarding their interaction and satisfaction with the video techniques on weeks 4 and 8 of the study. It is expected that students will enjoy MePlusMe’s personalized, friendly, and easy-to-use design, as well as the multimedia instructional videos alongside their supporting documents. We expect to see sufficient engagement with MePlusMe’s techniques as well as positive feedback about MePlusMe’s design and contents.

## Methods

### Recruitment and eligibility

Undergraduate and postgraduate students undertaking full- or part-time study at the host University were eligible for inclusion. Potential participants also had to be over 18 years old and comprehend English well enough to understand the intervention materials. They also had to present only minor to moderate, and not severe, psychological difficulties, as assessed using the GAD-7 [45], the PHQ-9 [46], and the Warwick-Edinburgh Mental Wellbeing questionnaire (WEMWB) [54].

Participants were recruited using various channels, including advertisements posted on the University’s online portal (blackboard), communications from the Student Union and social media. Leaflets about the study were also distributed around the campuses and at the end of some lectures. The University student support services and the student engagement team also provided information about the study to students where appropriate. Recruitment communications included basic information about the study, what it entailed, and the eligibility criteria. The study received ethical approval by the host institution (anonymised for the peer review process).

### Registration to the study

Whether they were recruited online or face-to-face, potential participants were given a link to a webpage where they could read detailed information about the study. They were also presented with the screening statements to decide whether alternative services would be more suitable to them and allow them to exit at this time point. Remaining participants were then presented with the consent form. They had to agree to all statements on the form and register themselves to participate in the study by leaving their contact details. They were then contacted by the research team within a few days of registering to invite them to the face-to-face group session. Each student had to attend a group session only once; twenty face-to-face sessions were offered in total.

## Materials

### The system

**Fig. 1:**
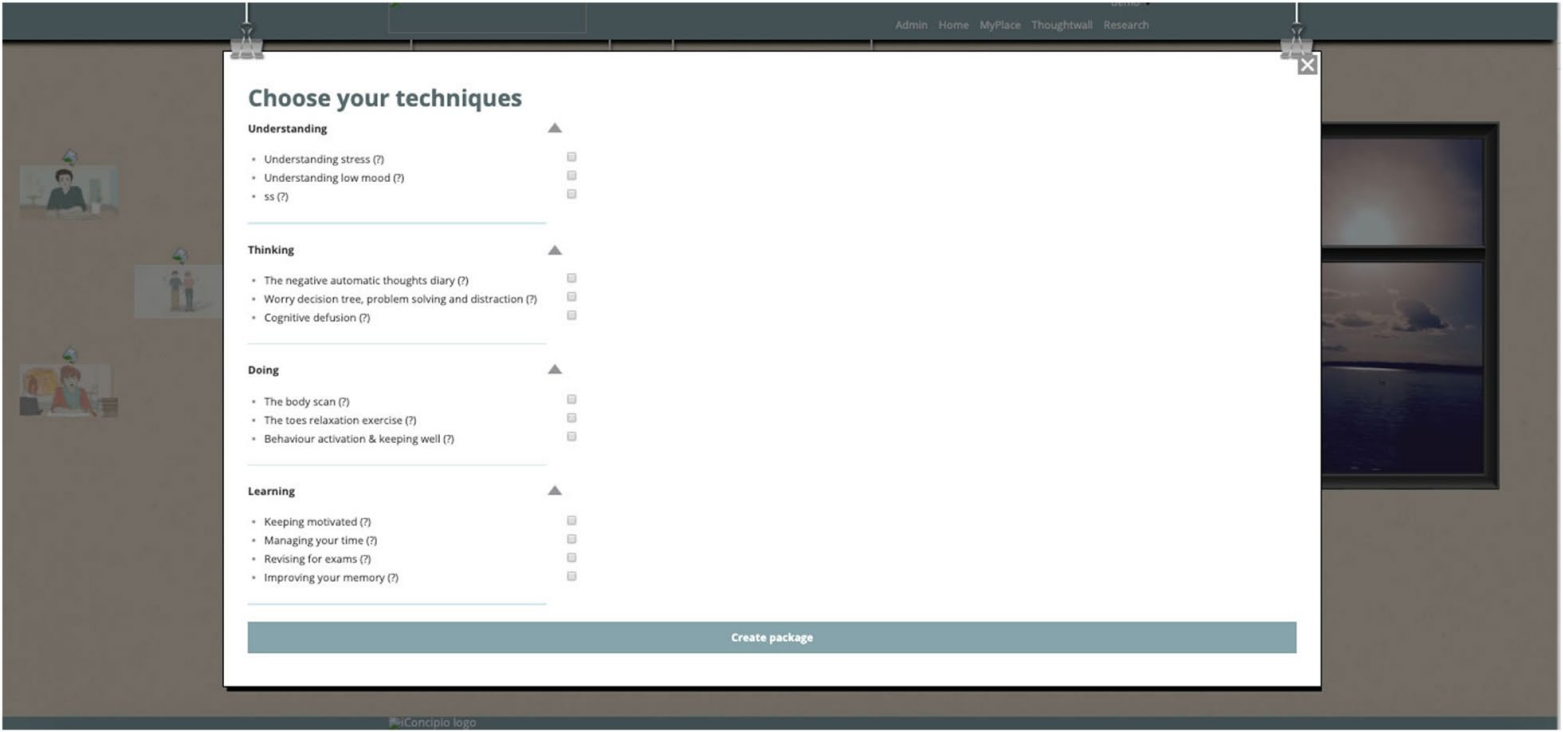
Screenshot of the Library route

**Fig. 2:**
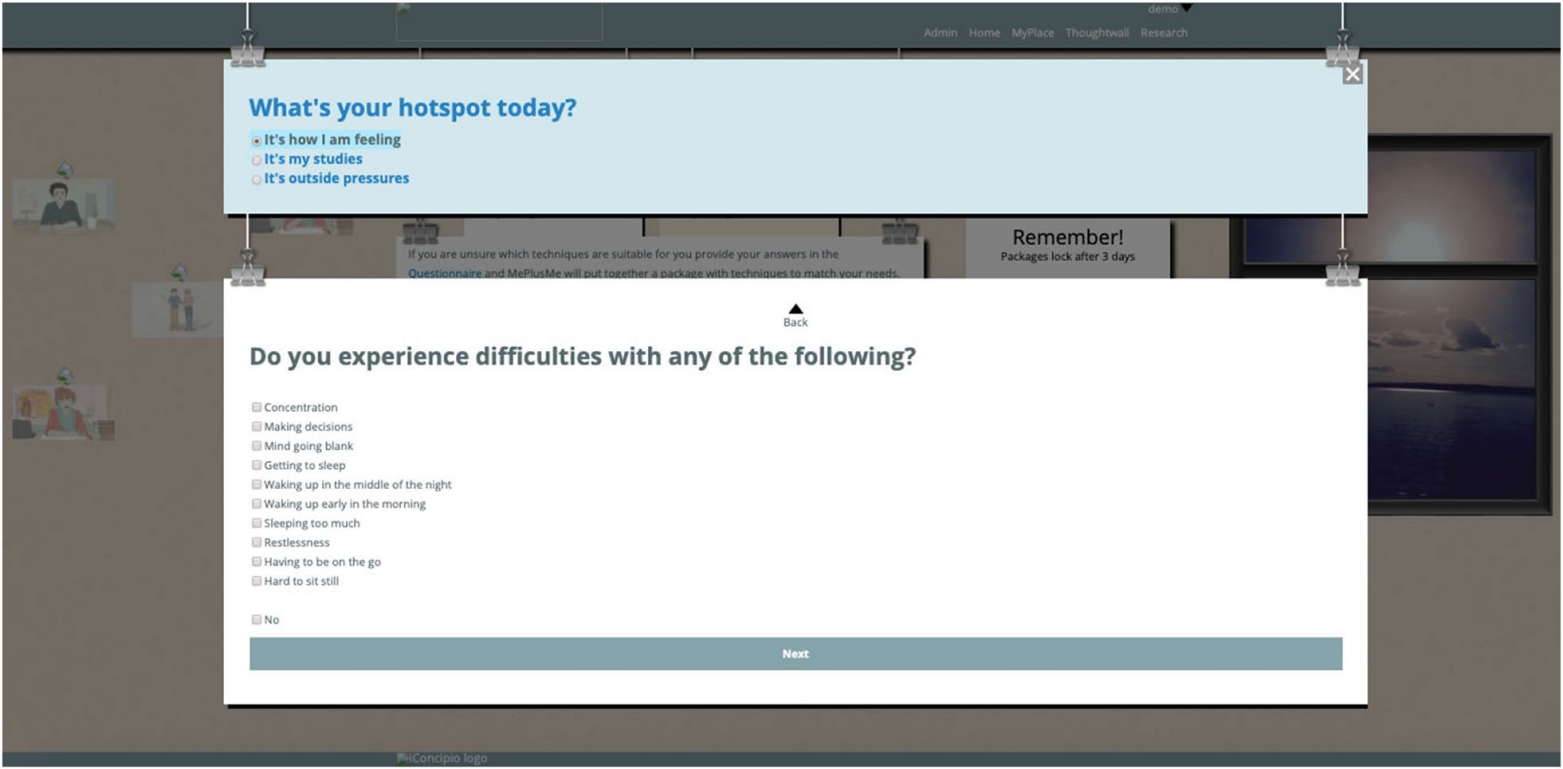
Screenshot of the Questionnaire route

MePlusMe, the online support system developed by iConcipio, the contents of which was under study here, offers access to techniques tailored to users’ needs and designed to address mild to moderate symptoms of anxiety and depression, as well as study-related difficulties. Following registration, users can either follow the symptoms-route (‘Questionnaire’) or the techniques-route (‘Library’), before they receive a tailor-made package with techniques that best address their needs at the time (see Figs. 1 and 2). Techniques are demonstrated using 2D animated videos. Users can then practise these techniques in their own time. A detailed description of the system can be found in the introduction. For the purposes of the present study, an offline demo version of the system was used.

### *Generalized Anxiety Disorder Scale* [GAD-7] [45]

The GAD-7 is used as a screening tool and a severity measure for generalised anxiety disorder. It comprises seven items and is scored using a four-point Likert scale ranging from ‘*not at all*’ (scored “0”) to ‘*nearly every day’* (scored “3”). The items are negatively framed, therefore higher scores indicate increasing symptoms. Possible scores range from 0 to 21, and scores of 5, 10, and 15 are taken as the cut-off points for mild, moderate, and severe anxiety.

### *Patient Health Questionnaire* [PHQ-9] [46]

The PHQ-9 assesses the severity of depressive symptoms. The scale includes nine statements scored using a four-point Likert scale ranging from ‘*not at all*’ (scored “0”) to ‘*nearly every day’ (scored “3”).* The items are negatively framed, therefore higher scores indicate increasing symptoms. Possible scores range from 0 to 27; scores ranging 0–4 indicate no depressive symptoms, 5–9 mild, 10–14 moderate, 15–19 moderately severe, 20–27 severe depressive symptoms.

### *Warwick*-*Edinburgh Mental Wellbeing scale* [WEMWB] [54]

The WEMWB scale was used to assess participants’ mental wellbeing. The WEMWB comprises 14 positively worded wellbeing statements and uses a five-point Likert scale ranging from ‘*none of the time*’ (scored “1”) to ‘*all of the time’* (scored “5”). Higher scores indicate enhanced mental wellbeing. Possible scores range from 14 to 70. England’s population mean score is 51.6 (SD = 8.70) (Health Survey for England. 2010; *n* = 7020).

PHQ-9, GAD-7, and WEMWB ratings were obtained at baseline and repeated at Weeks 2, 4, and 8. The absence of severe difficulties was also ascertained during the screening phase, where participants were presented with statements with regards to risky and/or aggressive behaviour, unusual sensory experiences or beliefs, and intentions of self-harm, and were prompted to think whether they relate to any of these experiences. Participants who related to any of these statements would automatically be given information on where to seek more appropriate help, including the accessible mental health helpline services SANE (http://www.sane.org.uk/) and Nightline (https://www.nightline.ac.uk/), and they would be excluded from the study. However, none of the participants screened out for this reason.

### *Academic Self*-*Efficacy scale* [ASE] [55]

The SEF was used to measure self-efficacy regarding study-related skills. The original scale was developed with U.S. college students and comprises 27 statements that describe positive study-related behaviours (e.g., taking good lecture notes) and uses a ten-point scale where 0 means ‘*not at all confident*’ and 10 means ‘*extremely confident*’. The scale was adapted with permission of its authors in two ways: (a) some items of wording were adapted to enhance comprehensibility for the UK setting, for example “term papers” were replaced by “coursework” and (b) two of the 27 items were removed (“Having more tests in the same week” and “Getting along with family members”) because they were of limited relevance to the study participants. We used a total self-efficacy score (rather than examining separate subscales), and so possible scores range from 0 to 250, with higher score denoting greater self-efficacy. Although this item removal will affect the psychometric properties of the measure, we consider this effect to be of limited importance because we are using a total (rather than sub-scale) score, and (because this is an exploratory feasibility study) using scale score only for descriptive analyses.

### Everyday Functioning

The users’ everyday level of functioning was assessed using the question “*How well are you getting on now in your daily life*”, which was measured using a five-points Likert scale ‘*not at all well’* to ‘*extremely well*’.

### System evaluation questions

At Baseline, participants were asked the reason they decided to participate in the study (possible responses to select all that apply: *“I am currently having a difficult time studying at university”, “I am currently having a difficult time emotionally at university”, “I think the support system (MePlusMe) is a great idea and I want to find out more”, “I have nothing better to do”, “Other, please specify”*). They were moreover asked how satisfied they were with the features and the contents of MePlusMe (i.e., Questionnaire, Library, MyPlace, the video techniques) with response options including *“Very poor”. “Poor”, “Fair”, “Good”, “Very Good”,* what they liked and did not like about the overall design of the system (open-ended questions), whether they liked the name (*“Like a lot”* to *“Dislike a lot”*), whether they liked the “MePlusMe Philosophy” video (*“Like a lot”* to *“Dislike a lot”*), whether they used other online support systems (e.g., self-help websites, MoodGym, Living Life to the Full) and how they compare to MePlusMe, as well as their overall satisfaction of the system (*“Very satisfied”* to *“Very dissatisfied”*). They were also asked to comment on areas for improvement.

At Weeks 4 and 8, participants were asked how often they watched (*“Everyday”, “1*–*3 times/week”, “1*–*3 times/month”, “Only once”, “Never”*) and practised the techniques (*“Μore than 3 times/week”, “1*–*3 times/week”, “1*–*3 times/month”, “Only once”, “Never”*), how they felt MePlusMe has helped them so far (open-ended), whether they contacted student support services after accessing the materials (*“Yes”/”No”*), where they will seek support in the future if need be (*“Approach Student Union only”, “Approach Student Union in addition to using MePlusMe”, “Only use MePlusMe”, “Not sure”, “Other*—*Please specify”,* their overall satisfaction with the system (*“Very satisfied”* to *“Very dissatisfied”*), how likely they are to recommend MePlusMe to a friend (*“Very likely”* to *“Very unlikely”*), and whether they plan to continue using the system after the completion of the study (*“Definitely will continue”* to *“Definitely won’t continue”*). They were also invited to share their recommendations to improve MePlusMe. Participants had the opportunity to provide qualitative feedback to questions, where appropriate.

### Procedure

The study was conducted over an 8-week period between October and January 2018, and included (a) an online session where students had the opportunity to read in depth information about the study, decide whether they meet the study’s inclusion criteria and sign the consent form, (b) a face-to-face group session (Βaseline), and (c) three online follow-up sessions at Weeks 2, 4, and 8. All data were collected online using the Qualtrics software.

#### Group session (baseline)

The aim of the group session was to introduce participants to the system and its contents, take them through the system’s assessment routes (Questionnaire or Library) and create the individual packages of techniques tailored to their needs. Most of the verbal information communicated on the day were coming from a script to keep the same conditions across participants and to replicate as closely as possible the experience of the fully developed online system (i.e., verbal instructions were given as they would see them on the system).

In the first part of the session, participants were introduced to the system by watching a video on the rationale of the system (MePlusMe’s “philosophy”, which is available online [56]) and were asked to browse the system offline to get a feel of how it looked and worked. They were then logged into the system and asked to navigate their way through it, use its available functionalities and access its contents. Baseline measures on their mental wellbeing (i.e., GAD-7, PHQ-9, and WEMWB), level of self-efficacy regarding study-related skills (i.e., ASE) ands demographic information were collected at this stage.

In the second part of the session, participants were individually administered by a member of the research team the assessment route they preferred (Questionnaire or Library) to identify their current difficulties. They were then asked how well they were functioning in their lives whilst experiencing these difficulties (Visual Analogue Scale question). Participants were then required to name their package of techniques and indicate whether they wished to receive reminders to watch the videos for the duration of the study. A 20-min break followed, during which researchers used the students’ answers to put together their individual personalised package of techniques, upload it in an individual folder on an online storage provider and email students a link to access their folder.

After the break, participants were asked to log into their emails and click on the link sent to them to access the folder. The folder contained their individual package of techniques in video format, a document explaining when to use each technique as well as, where applicable, the documents necessary for applying the techiques. Participants were invited to watch at least one video and familiarise themselves with the other video techniques before being asked to answer questions about their experience with the system until that point (see “System evaluation questions” for Baseline). At the end of the group session, participants were encouraged to watch the videos and apply the techniques in their own time. Participants were reminded to expect e-mails at Weeks 2, 4, and 8 to complete the follow-up measures. In addition, those who had opted to receive reminder emails, were told to expect these on Days 4, 8, 12, 22, and 45. All participants were reminded to expect e-mails at Weeks 2, 4, and 8 to complete the follow-up measures.

#### Follow-up online sessions

Participants were asked to complete questions assessing their degree of current functioning, measures pertaining to their mental wellbeing (i.e., GAD-7, PHQ-9, and WEMWB) and a self-efficacy questionnaire regarding study-related skill (i.e., ASE) at Weeks 2, 4, and 8. In addition, at Week 4 and Week 8 they were asked to answer questions regarding their engagement with the video techniques, potential after effects from their usage and their overall satisfaction with the system (see “System evaluation questions” Weeks 4 and 8). Participants who had not submitted their answers were sent a reminder 3 days after the date they were due to complete the online survey at weeks 2, 4, and 8.

### Analysis

All demographic and scale data and comment responses to open-ended questions were transposed from the MePlusMe system to Microsoft Excel and, following checking and appropriate coding, were entered into an SPSS (version 23) spreadsheet for descriptive analyses. The qualitative data collected via the open-ended questions in the questionnaires was analysed using Thematic Analysis [57], a common approach in qualitative research for identifying patterns of meaning (or “themes”) within data. The researchers scrutinised the written comments made by participants for patterns and categories and these are presented in tabular form below along with examples of the comments from which the themes were derived.

## Results

### Participant characteristics

Participants were ten undergraduate and three postgraduate students at the multicultural, ‘post 1992’ University in London (University of West London), a UK HEI. As may be seen in Table 1, nine of the thirteen participants were female, the mean age was 31.3 years (SD = 10.25), with female participants younger by nearly 8 years than their male counterparts. Seven were White, one was African, one Caribbean, one Asian, and one mixed (White/Black African).

**Table 1:** Participants characteristics (GAD-7: Generalized Anxiety Disorder Scale, PHQ-9: Patient Health Questionnaire, WEMWBS: Warwick-Edinburgh Mental Wellbeing scale)

Gender	*n*	Age (years)		Status			Year of study			Baseline measures mean (SD)		
		Mean	SD	Home	EU	Int/l	1st	2nd	3rd	GAD-7	PHQ-9	WEMWB
Female	9	28.8	9.6	4	2	3	6	2	1	8.1 (2.7)	9.7 (6.2)	45.0 (9.7)
Male	4	36.5	10.6	3	1	0	1	1	2	6.0 (3.8)	5.3 (2.9)	48.3 (9.3)
Total	13	31.3	10.2	7	3	3	7	3	3	7.5 (3.1)	8.3 (5.7)	46.0 (9.3)

The responses to scaled questions indicated that these data were sufficiently normally distributed for mean and standard deviation values to be meaningful descriptors: skewness statistics for all baseline findings were found to be between − 0.5 and + 0.5, and the Shapiro–Wilk test was non-significant for all scale measurements.

### Reasons for participating in the study

Out of the 13 initial participants eight reported that they are *“currently having a difficult time studying at university*”, four reported that they are “*currently having a difficult time emotionally at university”*, seven that “*the support system (MePlusMe) is a great idea and I want to find out more”,* and two also selected “*other”*, explaining that they “*want to improve coping techniques*” and that *“I am interested in how things happen and why*”.

### Progress and completion

As shown in Table 2, out of the 13 initial participants, seven completed the follow-up measures in Week 2, five in Week 4 and six in Week 8 (Week 8 attrition rate: 53.85%). The study was not powered to determine the effectiveness of the intervention, but all participants reported decreased symptoms of anxiety and depression and increased (Weeks 2 & 4) or stable (Week 8) wellbeing scores, compared to the Baseline. With regards to the ASE scores (see Table 2), a general improvement in scores was evident over the successive ratings, with all four of the participants who completed self-efficacy ratings at Week 8 having improved scores compared to their baseline ratings. Similarly, the VAS (see Table 2) scores showed an improvement at successive time-points to Week 4, with a modest reduction in Week 8.

**Table 2:** Mean mental wellbeing score changes over 8 weeks (standard deviation of scores in parenthesis) (GAD-7: Generalized Anxiety Disorder Scale, PHQ-9: Patient Health Questionnaire, WEMWBS: Warwick-Edinburgh Mental Wellbeing scale)

	Baseline	Week 2	Week 4	Week 8
GAD7	7.5 (3.1)	3.1 (1.6)	2.2 (3.3)	3.3 (3.8)
PHQ9	8.3 (5.7)	4.1 (3.7)	4.4 (5.6)	4.7 (4.0)
WEMWBS	46.0 (9.3)	54.4 (8.5)	48.4 (13.7)	47.5 (12.2)
n	13	7	5	6
Academic self-efficacy	151.1 (43.1)	169.4 (42.6)	181.2 (54.0)	178.5 (61.2)
n	13	7	5	4
Everyday functioning	2.38 (0.87)	2.71 (0.49)	3.60 (1.14)	3.33 (0.52)
n	13	7	5	5

### System evaluation

#### Impressions of the MePlusMe video that explains the rationale behind the system

Participants reported both positive and negative impressions of the first MePlusMe video that they watched at Baseline, which explains the rationale behind the system (MePlusMe’s philosophy [56]). Positive comments included comments on its style and good design, as well as the ease of understanding and the non-judgemental content of the message. Negative comments centered around the fact that the video was not informative enough, the fact that the basic style was unengaging, and changes needed in the voice-over. Some of the comments are listed below and can be seen in Tables 3 and 4.

**Table 3:** Positive impressions of first MePlusMe video

Theme	Participant comment examples
Style/well-designed	It is design simple and efficient. Communicate in a good way
	The video and animations were well done
	The way it was styled and presented was good
	The use of visual and audio
Ease of understanding	It simple to understand/it is a great and easy way to start improving yourself
	I like story boards, it is a very clever way of transferring information. The audio is at a good pace and tone
	The video did a good job at explaining the program
Content of message	I liked the mention that tendencies are your tendencies and are neither good nor bad

**Table 4:** Negative impressions of first MePlusMe video

Theme	Participant comment examples
Problems understanding message	I thought the video could have been more informative. It could have explained a bit more about techniques etc
Basic style unengaging	Simple animation—not so eye catching and a bit difficult to relate to
	Lack of ethnic diversity in video
Choice of voice-over	Interesting concept, but could have chosen another voice actor
	The way it was styled and presented was good, only felt that the voice over should be sped up a little

#### Engagement with MePlusMe video techniques after first viewings

After first viewing the MePlusMe videos there was limited subsequent engagement, with the two major factors identified were the fact that no re-watching was needed and time constraints. Some of the comments are listed below and can be seen in Table 5.

**Table 5:** Reasons for reduced engagement with MePlusMe videos after first viewings

Theme	Participant comment examples
Already incorporated techniques	I tried to use them in my own style, so I watched them a few times and then I used them according to my lifestyle
	After watching once, I didn’t need to re-watch the videos I just put the techniques into action and when I did view them it was to refresh and make sure I understood
	Many of the techniques did not really provide benefit from being repeated. I got lots of helpful advice for how to prepare for exams, and don’t feel that I would benefit from watching the videos again
Time constraints	Due to time
	I’ve been busy

#### How has MePlusMe helped participants so far—positive outcomes

Participants generally expressed that their engagement with the MePlusMe techniques was a positive experience. No specifically negative outcomes were identified. The major positive outcomes identified were improved positive thinking and enhanced memory, improved self-confidence, reassurance that self-improvement is possible, reassurance that others had similar experiences, practical outcomes through study tips. Some of the comments are listed below and can be seen in Table 6.

**Table 6:** Positive outcomes from engaging with MePlusMe

Theme	Participant comment examples
Improved positive thinking and enhanced memory	I tried to replace my negative thoughts with positive ones
	It has enhanced my memory
Improved self-confidence	I feel confident about myself and the place I am right now
Provided reassurance that self-improvement is possible	It made me feel better knowing there were more things I could be doing to help myself
Provided reassurance that others had similar experiences	Assured me that everyone is going through the same issues and also with the right thinking about them and solving them correctly
Provided practical outcomes through study tips	I managed to have a pretty clear schedule
	Good tips for planning exam studying in January
	Good study tips
	I like how it has a pragmatic approach to actually giving you some techniques and work sheets which are tangible

#### Evaluation of the features and contents

A high level of satisfaction with the Questionnaire was indicated. As can be seen in Table 7, the extent of endorsement of the particular features was consistently high, ranging between 69% for a single aspect (the clarity of wording within the Library), to 100% for several elements (the video techniques as a whole; the layout and the phrasing within MyPlace).

**Table 7:** Likert scaled responses concerning the MePlusMe system

	Participants rating ‘good’ or ‘very good’	% rating ‘good’ or ‘very good’
Questionnaire		
Layout/navigation—ease of use	10/13 (77%)	77
Clarity of the wording/phrasing,	12/13 (92%)	92
Usefulness	11/13 (85%)	85
Specific features (e.g., MyPlan)	12/13 (92%)	92
Overall	12/13 (92%)	92
Library		
Layout/navigation—ease of use	11/13 (85%)	85
Clarity of the wording/phrasing	9/13 (69%)	69
Usefulness	10/13 (77%)	77
Specific features (e.g. MyPlan)	11/13 (85%)	85
Overall	11/13 (85%)	85
MyPlace		
Layout/navigation—ease of use	13/13 (100%)	100
Clarity of the wording/phrasing	13/13 (100%)	100
Specific features (e.g. MyMessages	12/13 (92%)	92
Overall	12/13 (92%)	92
Video techniques		
Illustrations	10/13 (77%)	77
Narrator’s voice	11/13 (85%)	85
Clarity of the study board	12/13 (92%)	92
Ability to sustain interest	11/13 (85%)	85
Overall	13/13 (100%)	100

In addition to the above, participants reported that the reasons behind their likely engagement in the future include the professional design, their confidence that using the system will continue to be helpful and the fact that the techniques were useful. It was also reported that the participants may use the techniques, without having to re-watch again the videos. When asked if they would recommend MePlusMe to others, they reported positively and their reasons for doing so include a sense that others would also potentially benefit, particularly if the scope of the information is expanded and the fact that the advice provided is pragmatic and useful. Suggestions for improving MePlusMe include its development into an app form and the inclusion of an interactive forum that would allow users to engage directly with professionals. The overall impression of MePlusMe’s system was positive and most users were satisfied, as the system was found to be useful and practical, calming, relaxing, and easy to use and navigate through, having a clear and attractive design, being engaging as well as bespoke and tailored to the users needs. Most used the techniques 1–3 times a week or more. Participants were uncertain whether they liked the name “MePlusMe” “a lot” or “a little” with equal numbers of responders selecting these alternatives) and that they had not used other online support systems in the past (10/13 responders, 77%). None of the participants approached the student support services in the duration of the study, most reported that they will continue to use MePlusMe after the completion of the study, and most of participants claimed that if they will seek support in the future, if need be, they will do so by approaching the student union in addition to using MePlusMe. Negative impressions were also identified and were invariably related to the design and user experience. Specifically, participants reported ambiguity in the wording of the questionnaire used to assign the techniques, videos being too long but also too short and the fact that the material covered is accessible elsewhere. Table 8 presents the aspects of MePlusMe that participants reported to like and dislike most.

**Table 8:** Aspects of MePlusMe which participant said they most liked and disliked

Most liked	Most disliked
Friendly tone	Not new—other similar apps
Good functionality/ease of navigation	Not engaging visually
Ease of use	Poor navigation
Visually appealing	Limited in content/focus
Informative	
Personalised aspect	
Interactive features	
Library component	

## Discussion

The present study investigated the feasibility, and acceptability of the contents, design, and functionalities of MePlusMe. This investigation took place before the system’s online functionalities are fully developed in order to inform further development. Responses included positive remarks as well as useful suggestions for the improvement of the system. MePlusMe was found to be practical, easy to use, engaging, and tailored to users needs, but also in need of enrichment of content and of using more diverse characters. Moreover, the questionnaire and library routes that were provided to the users and the subsequent steps (e.g., responding to the VAS scale) were successfully navigated by users, showcasing the feasibility of our approach.

When participants watched their first video describing one of the techniques, they commented on its simple, professional, and efficient design, as well as on its good style and presentation. Moreover, they found the contents easy to understand and non-judgemental. While some participants found that the audio had a good pace and tone, others considered it to be unengaging and suggested changes in the voice-over, such as speeding it up and using a different voice actor. A lack of diversity and an ambiguity in the wording of the questionnaire were also noted as was the simplicity of the animation and the fact that is was not engaging visually. Participants were divided when it came to whether they liked the name “MePlusMe” or not. Further engagement with MePlusMe videos was limited, as participants found that not many repetitions were needed due to the contents being easily understandable, but also due to time constraints. Overall, the techniques offered by MePlusMe attracted positive comments that included improvements in positive thinking, memory, and self-confidence as well as reassurance that self-improvement is possible and that others have similar experiences. The study skills techniques were considered to provide a pragmatic approach and useful tips. Suggestions for further improvement were also collected. These included the system’s further development into an app and the option of having direct online engagement with professionals via a forum built into the system.

The comments and suggestions made by the participants provide a valuable source of information for the improvement of the system. We will keep developing and adding animated video techniques onto the system. This will give us the opportunity to introduce more diverse characters so that all students will feel that they can relate to a character. Also since videos will vary in length this will even out issues with regards to comments that videos are too long or too short. We will also introduce new narrators so that different voices can appeal to our target group. Moreover, the development of an app, through which users will be able to use the system via their smartphones, is also pending. In addition, the possibility of having direct contact with professionals through the system is already being considered and will take place through emails, video-conferencing and offline face-to-face treatment. Limitations of the present study include the small number of participants (*n* = 13), which though adequate for qualitative [58] and feasibility evaluation, does limit the interpretation of the measure scores to a descriptive analysis. Additionally, this study took place offline, rather than online which will be the standard mode of delivery when the system is fully developed.

When the online functionalities of the system are fully developed a feasibility study will follow, for which the protocol has been already published [56]. This study will allow for the assessment of the extent of change between pre- and post-intervention time points, with regards to mood, wellbeing, academic self-efficacy, and everyday functioning. Upon the completion of the feasibility study, a Randomized Controlled Trial (RCT) will ensue, in order to systematically measure the effectiveness of the intervention. Of note, of the six online support systems recently identified as having been designed specifically for HE students [42], only MePlusMe has conducted development studies, such as the current one, as well as an initial survey of end-users [52] and a proof-of-concept study [53]. This way MePlusMe responds to the call for more research on such systems [42].

Once the system is live, students will be able to use it anonymously, at their own space and time, as soon as symptoms arise and as often as they wish, removing common barriers in help seeking [59–61]. This will allow students to feel empowered by taking control over their mental health and personal effectiveness. It will further reinforce their motivation to change as well as their confidence and help them develop long-lasting coping skills. The online system will further host a space where the community of users will be able to provide additional support to each other. This build-in community could further help normalize the experiences of the users and give them a sense of belonging. The online nature of the system is by default holistic and inclusive. Therefore, the system will be available to students who would not normally seek face-to-face support. Furthermore, support will be tailor-made to the needs of each user every time they choose to use the system.

In addition to the benefits that the users will enjoy, HEIs could optimize their resources should they include online support in their range of services. Systems such as MePlusMe can indeed assist student support services to focus on cases presenting with severe difficulties, which are more pressing and require face-to-face contact. At the same time, MePlusMe can be offered to all students who present with mild or moderate difficulties, as a complementary service alongside the offline services. This way, student support services will emphasize on prevention, in a cost-effective manner [62]. Moreover, the system can be used as a first line of support for students on the waiting line to see a professional or as a fall-back plan, after therapy has been completed. MePlusMe could further provide analytics on students’ wellbeing and academic competence, which could allow HEIs to respond to students needs in a targeted manner. This way, HEIs will be in an informed position to improve student experience, reduce drop-out rates, and achieve higher ratings, intake, and income (for a more detailed discussion on the benefits of online support systems designed for students in HE see [42] and [43]).

## Conclusions

MePlusMe, is a multimedia, online system aiming to provide personalized support to HE students facing mild to moderate mental health difficulties as well as study skills difficulties and students who just want to take care of their mental health and improve their academic competence. It is the first system that is designed specifically for HE students that tackles both potential sources of problems, psychological and academic, in a personalized manner. Following a rigorous development process which includes a market research project with counsellors in HEIs, an initial survey of end-users [52] and a proof-of-concept study [53], this paper explored the acceptability and feasibility of the system’s developed contents, design, and functionalities, before its online functionalities are fully developed using qualitative data. Responses were overall positive, with useful comments and suggestions for further improvement. A larger feasibility study of MePlusMe’s design and contents using both quantitative and qualitative measures is currently underway in Riga Stradins University in Latvia. A feasibility study with a fully functioning system is the next step in the system’s development [63], which will be followed by an RCT.

## Data Availability

The full data set is available at request.

## Abbreviations

ADHD: Attention Deficit Hyperactivity Disorder
ASE: Academic Self-Efficacy
CALM: Computer Aided Lifestyle Management
CBT: Cognitive Behavioural Therapy
GAD-7: 7-Item Generalized Anxiety Disorder Scale
HADS: Hospital Anxiety and Depression Scale
HE: Higher Education
HEI: Higher Education Institution
HESA: Higher Education Statistics Agency
M.I.N.I: The Mini International Neuropsychiatric Interview
PHQ-9: Patient Health Questionnaire
RCT: Randomized Controlled Trial
RCP: Royal College of Psychiatrists
NHS: National Health Services
NUS: National Union of Students
UK: United Kingdom
WEMWB: Warwick-Edinburgh Mental Wellbeing questionnaire/scale
WHO: World Health Organisation
WMH-CIDI: World Health Organization World Mental Health Composite International Diagnostic Interview
